# Transcripts related with ammonium use and effects of gibberellin on expressions of the transcripts responding to ammonium in two invasive and native *Xanthium* species

**DOI:** 10.3389/fpls.2022.1035137

**Published:** 2022-10-26

**Authors:** Chang Zhang, Jia-Jun Luo, Jing-Bo Zuo, Zheng Zhang, Shi-Ting Wang, Xiao-Jia Zhang, Tian-Si Fu, Yu-Long Feng

**Affiliations:** Liaoning Key Laboratory for Biological Invasions and Global Changes, College of Bioscience and Biotechnology, Shenyang Agricultural University, Shenyang, Liaoning, China

**Keywords:** ammonium, assimilation, gibberellin, transcriptome analysis, uptake

## Abstract

Soil nitrogen forms are important for exotic plant invasions. However, little effort has been made to study the molecular mechanisms underlying the utilization of different N forms in co-occurring invasive and native plants. The invasive plant *Xanthium strumarium* prefers nitrate relative to ammonium, and mainly invades nitrate-dominated environments, while it co-occurring native congener *X. sibiricum* prefers ammonium. Here, we addressed the genetic bases for the interspecific difference in ammonium use and the effects of gibberellin (GA). Twenty-six transcripts related with GA biosynthesis and ammonium utilization were induced by ammonium in *X. sibiricum*, while only ten in *X. strumarium* and none for ammonium uptake. *XsiAMT1.1a*, *XsiGLN1.1* and *XsiGLT1b* may be crucial for the strong ability to absorb and assimilate ammonium in *X. sibiricum*. All tested transcripts were significantly up-regulated by GA1 and GA4 in *X. sibiricum*. *XsiGA3OX1a*, which was also induced by ammonium, may be involved in this regulation. Consistently, glutamine synthetase activity increased significantly with increasing ammonium-N/nitrate-N ratio for *X. sibiricum*, while decreased for *X. strumarium*. Our study is the first to determine the molecular mechanisms with which invasive and native plants use ammonium differently, contributing to understanding the invasion mechanisms of *X. strumarium* and its invasion habitat selection.

## Introduction

Nitrogen (N) is an essential component of proteins and nucleic acids, and thus plays critical roles in plant growth and development (Feng et al., 2009; Xu et al., 2012). In many ecosystems, N is an important limiting environmental resource for plants (Thomas et al., 2015). The two forms of inorganic N, i.e., nitrate and ammonium, are the main N source for plants in most ecosystems, and different plants prefer to use different N forms (Zhang et al., 2018; Sun et al., 2021). Rice (*Oryza sativa* L.), potato (*Solanum tuberosum* L.), tea trees (*Camellia sinensis* L.) and invasive plant *Flaveria bidentis* prefer ammonium, while wheat (*Triticum aestivum* L.), maize (*Zea mays* L.), cotton (*Gossypium hirsutum* L.) and invasive plant *Ipomoea cairica* prefer nitrate (Li et al., 2013; Huangfu et al., 2016; Chen and Chen, 2019). Our previous study showed that *Xanthium sibiricum* grows better in ammonium relative to nitrate, while its invasive congener *X. strumarium* grows better in nitrate treatment (Luo et al., 2022). However, the molecular mechanism underlying the better growth of *X. sibiricum* in ammonium is still unknown.

Plants absorb ammonium by ammonium transporter (AMT). *AtAMT1.1*, *1.2*, *1.3* and *1.5* are highly expressed in roots and all involved in ammonium uptake (Gazzarrini et al., 1999; Yuan et al., 2007). Ammonium uptakes were significantly reduced in the mutants of *atamt1.1*, *1.2* and *1.3* (Kaiser et al., 2002; Loque et al., 2006; Yuan et al., 2007). Before being used to synthesize organics, ammonium is firstly assimilated into glutamine and glutamate by glutamine synthetase (GS) and glutamate synthase (GOGAT), respectively. *AtGS1* and *AtGS2* were highly expressed in roots and shoots, respectively (Ishiyama et al., 2004). *AtGLN1.1*, one of *GS1* subfamily members, was more highly expressed under N starvation than other members of *GS1* subfamily. *AtGLN1.2* was induced by ammonium and more highly expressed under 10 mmol L^-1^ ammonium treatment than other members of *GS1* subfamily. The GS activity of AtGLN1.3 was stronger than that of other members of GS1 subfamily (Ishiyama et al., 2004). The GS activity was reduced in the mutant of *AtGLN1.2* which had low ammonium assimilation ability (Lothier et al., 2011). For GOGAT in *Arabidopsis*, all family members are involved in ammonium assimilation (Li et al., 2017). *AtGLU1* was highly expressed in leaves, while *AtGLU2* and *AtGLT1* were highly expressed in roots (Coschigano et al., 1998; Lancien et al., 2002).

Plant hormones can regulate utilization of different N forms (O'Brien et al., 2016). It has been demonstrated that nitrate can induce expressions of some cytokinin biosynthesis related genes, including *IPT3* (isopentenyl transferase 3) and *CYP735A* (encoding a cytochrome P450 monooxygenase), promoting cytokinin synthesis (Takei et al., 2004; Wang et al., 2004; Sakakibara, 2006). In turn, cytokinin can induce expressions of some nitrate utilization related genes, including *NRT1.3* (low-affinity nitrate transporter) and *NIA1* (nitrate reductase) (Brenner et al., 2005; Kiba et al., 2005; Sakakibara et al., 2006). However, few studies determined the interaction between hormones and ammonium. Recently, Li et al. (2019) found that shoot ethylene content increased in *A. thaliana* under high level of ammonium. Compared with the wild type, the mutant of *EIN3*, which acts as a key regulator in ethylene signal pathway, can increase ammonium tolerance. Gibberellin GA3 was also found to improve ammonium uptake and utilization in rice by up-regulating expressions of *OsAMT1.1* and *OsGS1.2* (Li et al., 2018).

*Xanthium strumarium*, native to north America, is a noxious invasive plant in north China (Feng, 2020). It prefers to absorb nitrate relative to ammonium, and grows better in nitrate (Luo et al., 2022). Consistently, it often invades disturbed habitats with nitrate as dominant soil N form. In contrast, its co-occurring native congener *X. sibiricum* prefers to use ammonium, and grows better in ammonium (Luo et al., 2022). The difference in N form utilization between these *Xanthium* species may be associated with invasion success of the invader, and its invasiveness evolution under the background of global change such as N deposition. The ratio of nitrate-N to ammonium-N in the atmospheric deposition N increases gradually in recent years in China (Yu et al., 2019). Thus, it is important to elucidate the mechanisms underlying this difference. In this study, we explored the molecular mechanisms underlying the strong ability to absorb and assimilate ammonium for *X. sibiricum*. We hypothesize that (1) more transcripts related with ammonium uptake and assimilation such as *AMT*, *GS* and *GOGAT* are induced by ammonium, and/or more highly expressed in *X. sibiricum* than in *X. strumarium*; (2) the same for the transcripts related with syntheses of some hormones, and these hormones up-regulate expressions of some transcripts related with ammonium uptake and assimilation. Our study is the first one that explored the molecular mechanisms underlying the difference in response to different N forms between invasive and native plants.

## Materials and methods

### Materials

The invasive plant *Xanthium strumarium* and its native congener *X. sibiricum* were used in this study. The seeds of these species were collected in Gaomabao village, Faku County, Shenyang City, Liaoning Province (42° 27′ N, 123° 0′ E; asl. 73 m), where no herbicides were applied to control the invader. Seed germination and seedling preparation were the same as in our previous study (Luo et al., 2022). Three similar-sized seedlings of each of the two species were grown separately in 1/2 Hoagland nutrient solution without N for 2 d, and then treated with 5 mmol L^-1^ KNO_3_ and NH_4_Cl, respectively. Four hour later, the fine roots of three seedlings of each species were collected separately for RNA sequencing (three biological replicates).

### RNA extraction and sequencing

The roots of the above-mentioned seedlings were collected for total RNA extraction using the MiniBEST universal RNA extraction kit (Takara, Dalian, China). The quality and quantity of the RNA extracts were assessed using 1% agarose gel electrophoresis, NanoDrop (Thermo Fisher Scientific, Inc., Waltham, USA.) and Agilent 2100 Bioanalyzer (Agilent Technologies, Palo Alto, USA), respectively. In order to acquire the full-length sequences of transcriptomes, PacBio sequencing (third-generation sequencing) was carried out firstly, as there was no genome data for the two *Xanthium* species. Then, HiSeq sequencing (second-generation sequencing) was performed in order to analyze the expression levels of the transcripts responding to different N forms. The sequencing methods were the same as in our previous study (Luo et al., 2022).

### Bioinformatics analysis

High-quality clean data were acquired using Cutadapt (version 1.9.1). The data were then assembled according to a method described by Grabherr et al. (2011). Transcript annotation was carried out based on non-redundant protein (NR), KEGG, GO and Swiss-Prot. Relative expression level (FPKM value) of each transcript was calculated using RSEM (v1.2.6). The expression difference in each transcript between ammonium and nitrate treatments was conducted for each species using the Bioconductor package DESeq (Li and Dewey, 2011). The transcripts were considered as differentially expressed transcripts (DETs) if their Benjamini and Hochberg (BH)-adjusted *P*-values (FDR) ≤ 0.05 and the magnitudes of the difference ≥ 2-fold (Luo et al., 2022). GO terms and KEGG pathway enrichment analyses were conducted for the DETs using GO-TermFinder and Kyoto Encyclopedia of Genes and Genomes (KEGG) database, respectively. Significant enrichments were also determined by BH-adjusted *P*-values (FDR) ≤ 0.05.

To determine the transcripts that may be responsible for efficient use of ammonium in the two *Xanthium* species, the expressions of the DETs belonging to *AMT*, *GLN*, *GLU* and *GLT* members were presented using heat maps, which were constructed using the pheatmap package in R (Costa et al., 2020). The differences in expressions of the DETs related with gebberalin (GA) metabolism were also analyzed, as GA may regulate expressions of *AMT*, *GLN*, *GLU* and *GLT* members. The methods had been described in detail in our previous study (Luo et al., 2022).

### Effects of gibberellin (GA) on expressions of related transcripts

Based on our results, GA was the main hormone significantly affected by N forms, and thus we determined its effects on expressions of the transcripts related with ammonium uptake and assimilation for *X. sibiricum*. The transcripts (to be measured) were determined based on our RNA sequencing data: expressed the most highly among the transcripts of each related gene family and induced by ammonium. In this study, the transcripts included an *AMT* member (*XsiAMT1.1a*), a *GLN* member (*XsiGLN1.1*) and a *GLT* member (*XsGLT1b*). The seedlings after N starvation (2 d) were treated for 10 h in the nutrient solution including 5 mmol L^-1^ NH_4_Cl with and without addition of 1 mg L^-1^ GA1 and GA4, respectively. Then, the fine roots were separately collected for measuring the relative expression levels of the transcripts using quantitative real-time PCR (three biological replicates). Quantitative real-time PCR was performed as described in reference (Luo et al., 2022). Primers were displayed in **Table S1**. The differences for these transcripts between GA treatments were tested using one-way ANOVA (SPSS Inc., Chicago, IL, USA).

### Effects of nitrogen forms on growth and glutamine synthetase

The seedlings of *X. sibiricum* and *X. strumarium* were treated separately with two ${\text{NO}}_{3}^{-}/{\text{NH}}_{4}^{+}$ ratios (nitrate-N: ammonium-N = 1: 9 and 9: 1) and two N levels (50% and 100% N concentrations of Hoagland nutrient) for 45 days. In this experiment, mixed N forms were used as the species, especially *X. strumarium*, could not grow well for long time in pure ammonium condition based on our preliminary observations. After treatment, recently matured leaves and fine roots were collected separately for each of six seedlings per species (six replicates), quickly frozen in liquid nitrogen, and stored in -80 °C for measuring glutamine synthetase activity, which was determined according to Li (2000). Six seedlings per species per treatment were harvested separately, oven dried at 60 °C for 3 d, and then weighed using an analytical balance. One-way ANOVA was used to test the differences among different N treatments for the same species, and independent sample *t*-test was used to analyze the differences between the two species under the same N treatment. The analyses were carried out using SPSS (Inc., Chicago, IL, USA).

## Results

### Overview of ammonium-responsive transcripts

**Figure 1:**
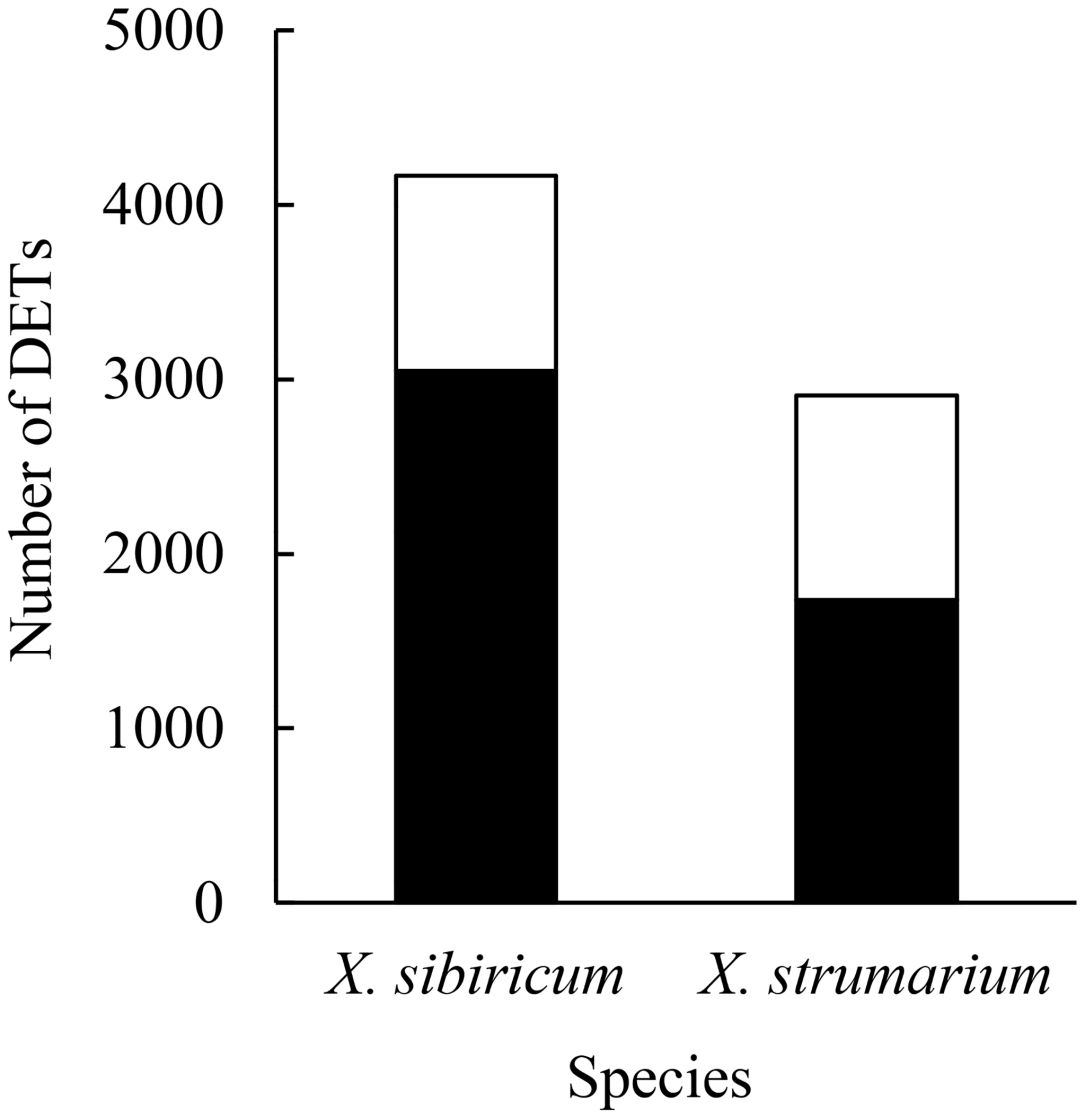
The numbers of differently expressed transcripts (DETs) between ammonium and nitrate treatments in *Xanthium sibiricum* and *X. strumarium*, respectively. Closed columns indicate the DETs induced by ammonium, i.e., expressed more highly under amonium relative to nitrate. Open columns indicate the DETs induced by nitrate.

The reads and measured bases ranged from 3.99 × 10^7^ to 6.38 × 10^7^ and from 5.99 × 10^9^ to 9.57 × 10^9^, respectively, and the Q30 values were all above 90% (**Table S2**), indicating that our mRNA sequencing results are valid. There were 4166 differentially expressed transcripts (DETs) between ammonium and nitrate treatments in the native plant *Xanthium sibiricum*, which were much more than those in the invasive plant *X. strumarium* (2907; **Figure 1**). In addition, much more DETs were induced by ammonium (expressed more highly under ammonium relative to nitrate treatment) in *X. sibiricum* than in the invader (3050 vs. 1737). In contrast, the proportion of nitrate-induced transcripts in total DETs was much higher in the invader than in *X. sibiricum* (40% vs. 27%), while the absolute numbers of the nitrate-induced DETs were similar for these two species (1170 vs 1116). GO terms analysis showed that the number of DETs enriched in molecular function was the largest, followed successively by those enriched in biological process and cellular component (**Figure S1**), suggesting that these two species responded to the conversion of different N forms mainly by changing molecular function. Most of the DETs of the two *Xanthium* species were enriched in catalytic activity, binding, metabolic process, and cellular process.

### KEGG pathway enrichment analysis of ammonium-responsive transcripts

**Figure 2:**
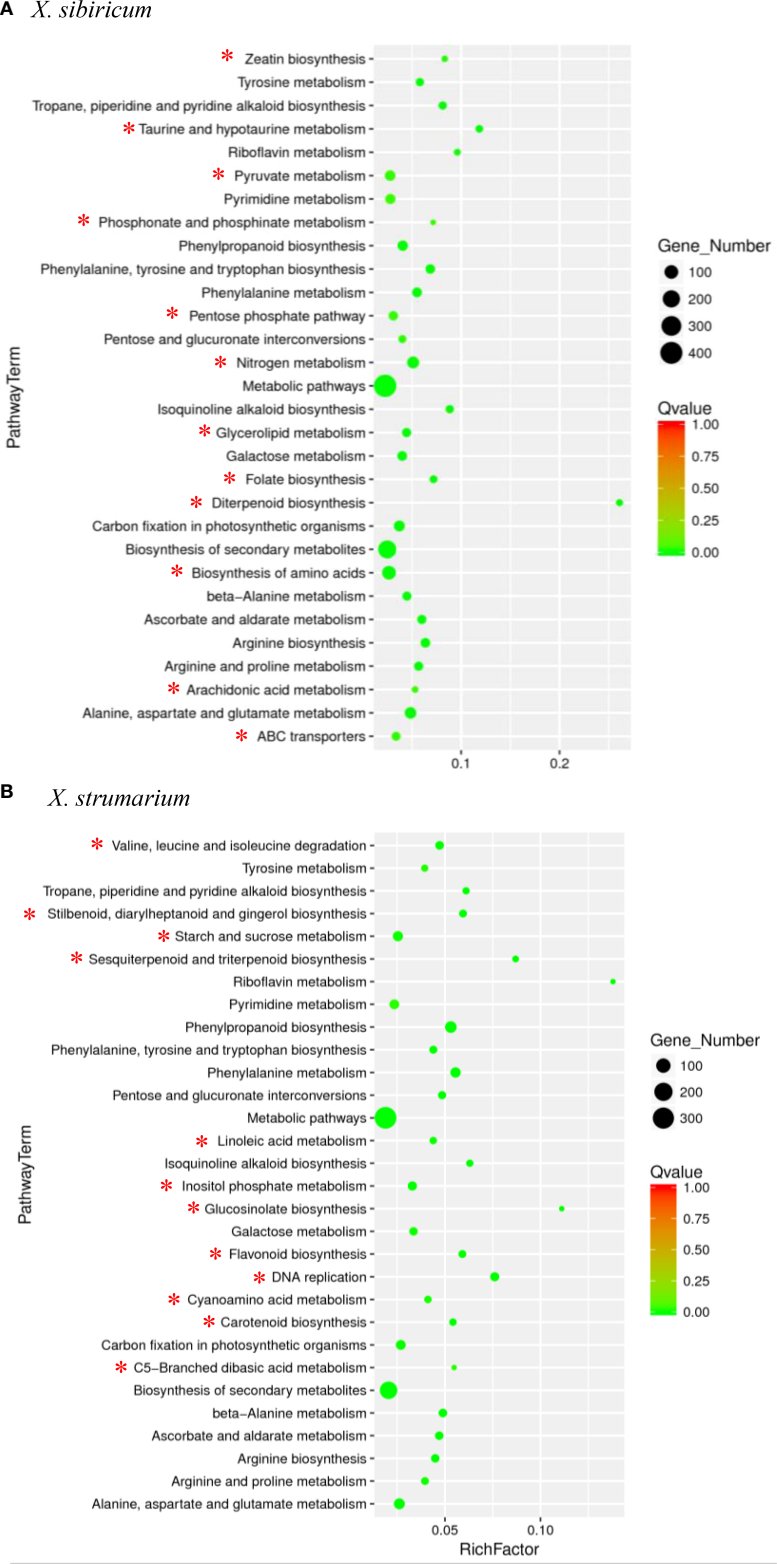
Pathways enrichment analysis for differently expressed transcripts (DETs) in *Xanthium sibiricum* **(A)** and *X. strumarium* **(B)**. Circle size indicates the number of DETs enriched in each pathway. Q value indicates significance of the enrichment, increasing from red to green. Rich factor represents the ratio of the enriched DETs to total transcripts in this pathway. The 12 unique pathways for each species are marked with *, and the 18 common pathways are not.

**Figure 3:**
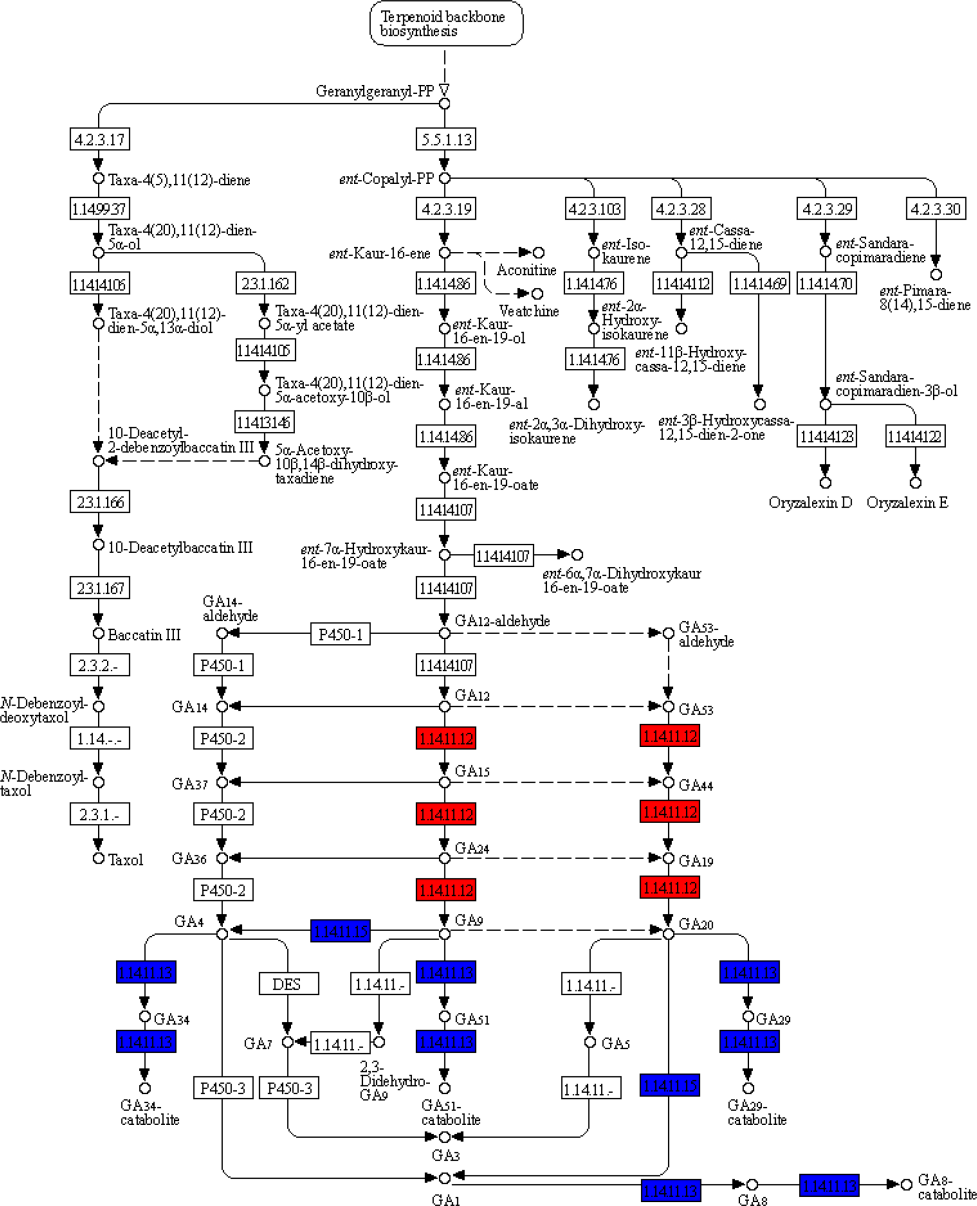
Differently expressed transcripts (DETs) of *Xanthium sibiricum* involved in GA biosynthesis and metabolism. Blue and red words represent that this kind of DETs were induced by ammonium and nitrate, respectively.

Among the top 30 pathways in which the DETs were significantly enriched for each of the *Xanthium* species, there were 18 common pathways for *X. sibiricum* and *X. strumarium*, and 12 unique pathways for each species (**Figure 2**). Among the unique pathways for each species, the numbers of the DETs enriched in biosynthesis of amino acids (followed by that in nitrogen metabolism), and starch and sucrose metabolism (followed by that in inositol phosphate metabolism) were the highest for *X. sibiricum* and *X. strumarium*, respectively. The ratio of the enriched DETs to total transcripts (rich factors) in diterpenoid biosynthesis (followed by taurine and hypotaurine metablism) and riboflavin metabolism (followed by glucosinolate biosynthesis) were the highest for *X. sibiricum* and *X. strumarium*, respectively. Interestingly, the DETs enriched in diterpenoid biosynthesis were involved in the biosynthesis and metabolism of gibberellin (GA) for *X. sibiricum* (**Figure 3**).

### Expression analysis of ammonium-use related transcripts

**Figure 4:**
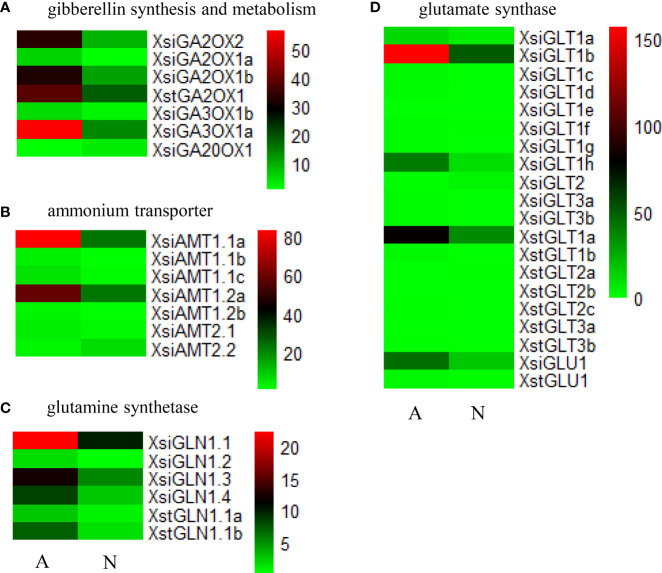
Expression patterns of the differently expressed transcripts (DETs) related with gibberellin synthesis and metabolism **(A)**, ammonium transporter **(B)**, glutamine synthetase **(C)** and glutamate synthase **(D)** in *Xanthium sibiricum* and *X. strumarium*. For *X. strumarium*, there were no DETs related with ammonium transporter. Different colors represent different expression levels (increasing from green to red). A and N indicate ammonium and nitrate treatments, respectively. The names of the transcripts were gased on our phylogenetic analyses (**Figures S2-5**).

In *X. sibiricum*, the transcripts of gibberellin 2beta-dioxygenase (GA2OX) [EC:1.14.11.13] and gibberellin 3beta-dioxygenase (GA3OX) [EC:1.14.11.15] were induced by ammonium, while the transcripts of gibberellin-44 dioxygenase (GA20OX) [EC:1.14.11.12] were induced by nitrate (**Figure 3**). In total, there were six DETs encoding these three enzymes in *X. sibiricum*: three for GA2OX, two for GA3OX and one for GA20OX (**Figures 4A**, **S2**). All these DETs were induced by ammonium except *XsiGA20OX1* which was induced by nitrate. Among the six DETs, *XsiGA3OX1a* was the most highly expressed (**Figure 4A**). For *X. strumarium*, however, only one DET (*XstGA2OX1*) related with GA metabolism were identified, which was induced by ammonium.

Seven *ammonium transporter* (*AMT*) transcripts, four *glutamine synthetase* (*GS*) transcripts and 12 *glutamate synthase* (*GOGAT*) transcripts were differently expressed in *X. sibiricum* between ammonium and nitrate treatments (**Figures 4B–D**, **S3-5**). Except *XsiAMT2.2* and *XsiGLT2* (induced by nitrate), all remaining 21 DETs of *X. sibiricum* were induced by ammonium. *XsiAMT1.1a*, *XsiGLN1.1* and *XsiGLT1b* were the most highly expressed among these differently expressed *AMT*, *GS* and *GOGAT* transcripts, respectively. For *X. strumarium*, however, no ammonium uptake related transcript and only 10 ammonium assimilation related transcripts (2 *GS* and 8 *GOGAT*) were differently expressed in ammonium and nitrate. All DETs of *X. strumarium* were induced by ammonium except *XstGLT2a*, which was induced by nitrate. The sequences of *XstGLT1a* and *XstGLT1b* were similar with that of *XsiGLT1b* (**Figure S5**), but their expression levels were lower than that of *XsiGLT1b* (**Figure 4D**).

**Figure 5:**
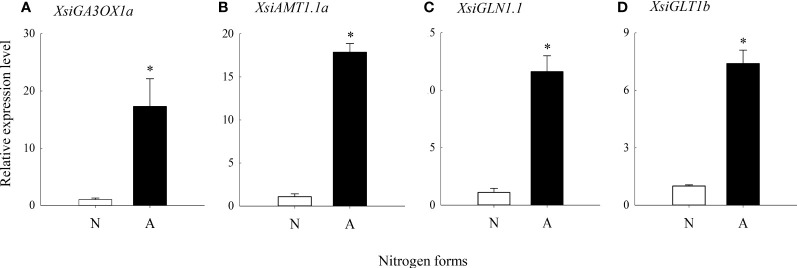
Differences in expression levels of *XsiGA3OX1a* **(A)**, *XsiAMT1.1a* **(B)**, *XsiGLN1.1* **(C)** and *XsiGLT1b* **(D)** between nitrate (N; open bars) and ammonium (**A**; closed bars) treatments based on our quantitative real-time PCR. *XsiActin1* was used as internal controls. Means + SD (*n* = 3). Asterisk indicates significant difference between ammonium and nitrate treatments (*P*< 0.05; independent sample *t*-test). The names of the transcripts were based on our phylogenetic analyses (**Figures S2-5**).

**Figure 6:**
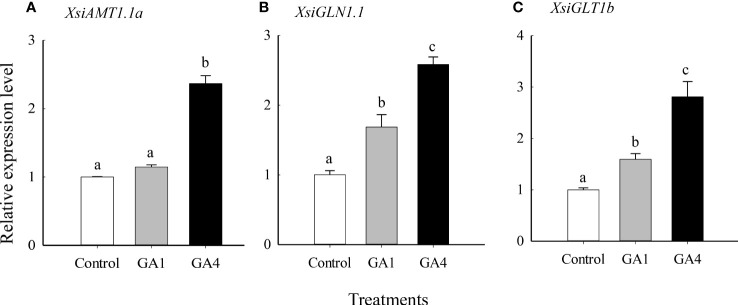
Effects of gibberellin (GA1 and GA4) on expression levels of *XsiAMT1.1a* **(A)**, *XsiGLN1.1* **(B)** and *XsiGLT1b* **(C)**. White bars, grown in nutrient solution including 5 mmol L^-1^ ammonium and without GA1 and GA4 addition; gray bars, grown in nutrient solution including 5 mmol L^-1^ ammonium and with 1 mmol L^-1^ GA1 addition; black bars, grown in nutrient solution including 5 mmol L^-1^ ammonium and with 1 mmol L^-1^ GA4 addition. *XsiActin1* was used as internal controls. Means + SD (*n* = 3). Different letters indicate significant difference between treatments (*P*< 0.05; one-way ANOVA). The names of the transcripts were based on our phylogenetic analyses (**Figures S3-5**).

Our quantitative real-time PCR also showed that *XsiGA3OX1a*, *XsiAMT1.1a*, *XsiGLN1.1* and *XsiGLT1b* were expressed much more highly in ammonium relative to nitrate treatment (**Figure 5**). Moreover, *XsiAMT1.1a*, *XsiGLN1.1* and *XsiGLT1b* were induced by GA1 and GA4 in ammonium, and the effects of GA4 were stronger than those of GA1 (**Figure 6**).

### Growth and glutamine synthetase activity

**Figure 7:**
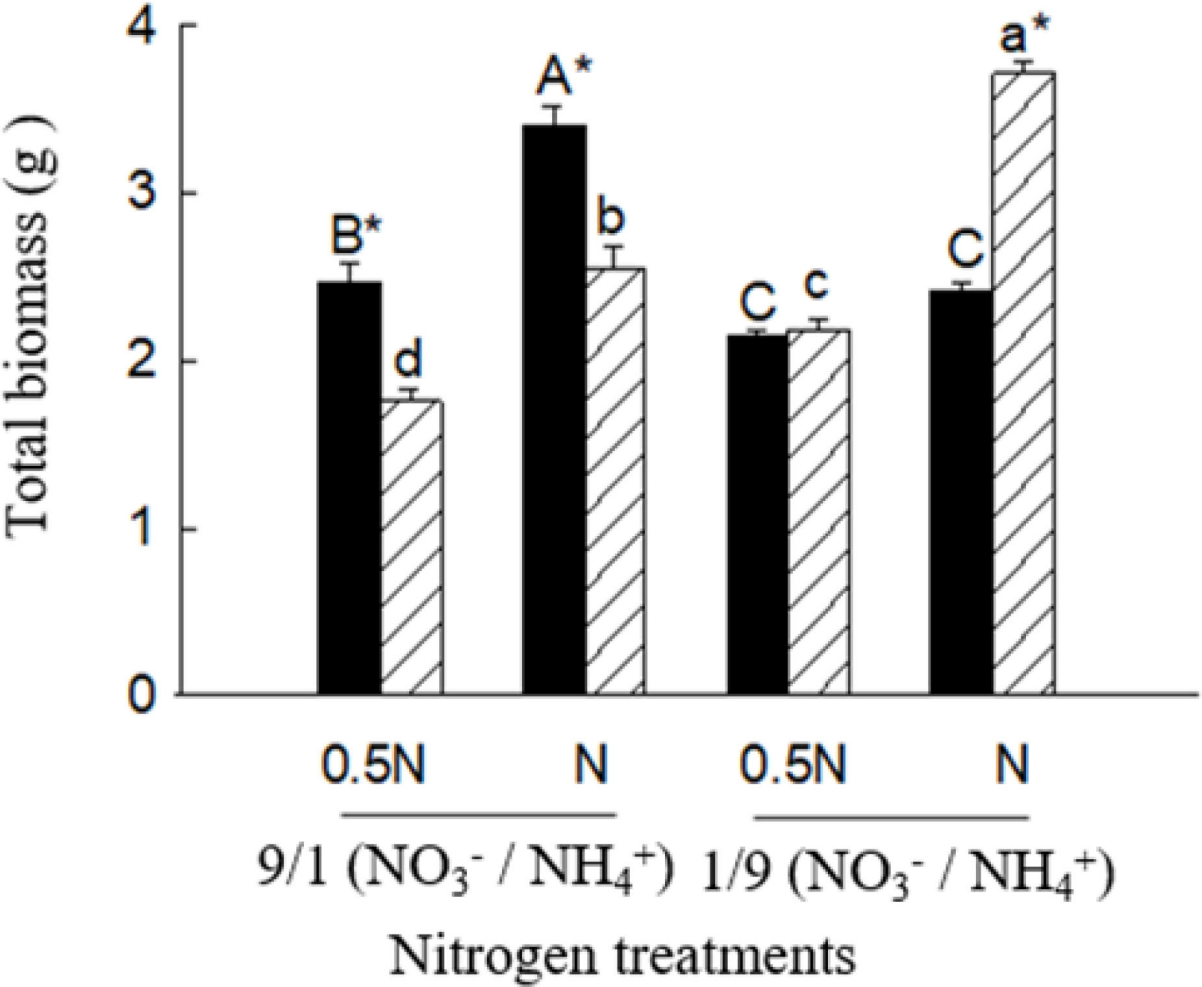
Total biomass of *Xanthium sibiricum* (striped bars) and *X. strumarium* (closed bars) in different nitrogen treatments. 0.5 N and 1 N indicate 50% and 100% nitrogen concentration of the Hoagland nutrient solution, respectively. Means + SE (*n* = 6). Different upper- and lowercase letters indicate significant dfferences among nitrogen treatments for *X. strumarium* and *X. sibiricum*, respectively (*P*< 0.05; one-way ANOVAs); * indicates significant difference between the invasive and native species in the same treatment (*P*< 0.05; independent sample *t*-test).

**Figure 8:**
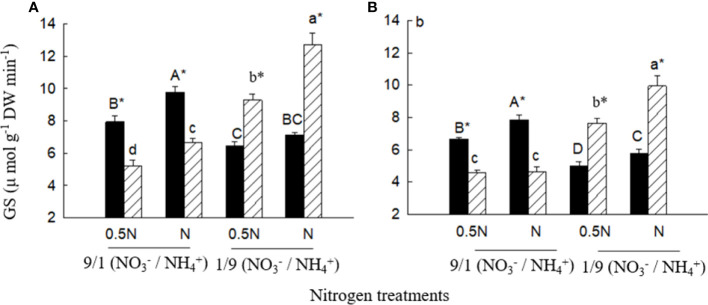
Glutamine synthetase (GS) activities in leaves **(A)** and roots **(B)** of *Xanthium sibiricum* (striped bars) and *X. strumarium* (closed bars) in different nitrogen treatments. 0.5 N and 1 N indicate 50% and 100% nitrogen concentration of the Hoagland nutrient solution, respectively. Means + SE (*n* = 6). Different upper- and lowercase letters indicate significant dfferences among nitrogen treatments for *X. strumarium* and *X. sibiricum*, respectively (*P*< 0.05; one-way ANOVAs); * indicates significant difference between the invasive and native species in the same treatment (*P*< 0.05; independent sample *t*-test).

Under the low ${\text{NO}}_{3}^{-}/{\text{NH}}_{4}^{+}$ ratio (1/9), total biomass (not significant in 0.5 N), leaf and root GS activities were significantly higher for *X. sibiricum* than for *X. strumarium*, regardless of N levels (**Figures 7**, **8**). Under the high ${\text{NO}}_{3}^{-}/{\text{NH}}_{4}^{+}$ ratio (9/1), however, the results were reversed. With decreasing ${\text{NO}}_{3}^{-}/{\text{NH}}_{4}^{+}$ ratio, *X. sibiricum* increased total biomass, leaf and root GS activities significantly, while *X. strumarium* decreased. Under the same ${\text{NO}}_{3}^{-}/{\text{NH}}_{4}^{+}$ ratio, total biomass (except *X. strumarium* under 1/9 of ${\text{NO}}_{3}^{-}/{\text{NH}}_{4}^{+}$ ratio), GS activities in leaves (except *X. strumarium* under 1/9 of ${\text{NO}}_{3}^{-}/{\text{NH}}_{4}^{+}$ ratio) and roots (except *X. sibiricum* under 9/1 of ${\text{NO}}_{3}^{-}/{\text{NH}}_{4}^{+}$ ratio) increased significantly with increasing N level.

## Discussion

Our results showed that many transcripts related with GA biosynthesis and metabolism, N uptake and assimilation were induced by ammonium in the native plant *Xanthium sibiricum*, and many of the transcripts related with N uptake and assimilation were up regulated by GA (**Figures 4**–**6**). These results indicate that in response to ammonium *X. sibiricum* may increase GA biosynthesis firstly, then increase its abilities of N uptake and assimilation, and thus growth (**Figure 8**). For the invasive plant *X. strumarium*, however, only nine N assimilation related transcripts were induced by ammonium, and no *AMT* transcripts were induced by ammonium (**Figures 4B–D**). In contrast, the relative quantity of nitrate-induced transcripts was much more in the invader than in *X. sibiricum* (**Figure 1**). All DETs enriched in DNA replication pathway are induced by nitrate for the invader (**Figure S6**), which indicates that nitrate may increase DNA replication and thus growth in the invader, providing molecular evidence for its strong ability to use nitrate. These results explain the reasons that *X. sibiricum* responded more strongly to ammonium, while the invader more strongly to nitrate (**Figure 7**; Luo et al., 2022). Exotic plants may be more like to invade the habitats where the dominant soil N form is what they prefer (Feng, 2020; Sun et al., 2021). *Flaveria bidentis* and *Ipomoea cairica* prefer ammonium and nitrate, respectively, and grow better in the habitats with related N forms as main soil N sources (Huangfu et al., 2016; Chen and Chen, 2019). Our study is the first one that determines the molecular mechanisms for co-occurring invasive and native plants to use different N forms, contributing to understanding the selection of invasion habitats of alien plants.

### Nitrogen uptake and assimilation

As our hypothesis, more transcripts related with ammonium uptake and assimilation (*AMT*, *GS* and *GOGAT*) were induced by ammonium in *X. sibiricum* than in *X. strumarium* (21 vs. 9; **Figures 4B–D**). Among the six ammonium-induced *AMT* transcripts, *XsiAMT1.1a* was the most highly expressed (**Figure 4B**). *XsiAMT1.1a*, *XsiAMT1.1b*, *XsiAMT1.1c*, *XsiAMT1.2a* and *XsiAMT1.2b* are clustered together with *AtAMT 1.1*, *1.2*, *1.3* and *1.5* in evolutionary tree (**Figure S3**), which all have ammonium uptake activities (Li et al., 2017). These transcripts (especially *XsiAMT1.1a* and *XsiAMT1.2a*) may be important for *X. sibiricum*’s higher uptake rate of ammonium relative to nitrate N, and thus faster growth in ammonium (Luo et al., 2022). Rice prefers ammonium relative to nitrate, which may be associated with its origin habitat (flooded wetland) where ammonium is the dominant N form (Xu et al., 2012), and many *AMT* members such as *OsAMT1.1* and *OsAMT1.2* were highly expressed and induced by ammonium (Sonoda et al., 2003; Li et al., 2016). Overexpression of *OsAMT1.1* improves ammonium uptake in rice, while the reverse is true for disruption of *OsAMT1.1* (Kumar et al., 2006; Li et al., 2016). However, *XsiAMT2.2* may not be important for *X. sibiricum* to absorb ammonium, as it was induced by nitrate (**Figure 4B**), and clustered together with *AtAMT2.1* in the evolutionary tree (**Figure S3**), which has not been found to have ammonium uptake activity (Li et al., 2017).

For *X. sibiricum*, *XsiGLN1.1*, *XsiGLN1.3* and *XsiGLN1.4* may all contribute to its fast ammonium assimilation and thus fast growth in ammonium, as they were highly expressed (especially *XsiGLN1.1*), induced by ammonium (**Figure 4C**), and clustered together with *AtGLN1.1* and *AtGLN1.2* in the evolutionary tree (**Figure S4**), which have been proved to have ammonium assimilation activity (Li et al., 2017). Coding genes of glutamine synthase were identified in many other species such as barley, rice, maize, *Arabidopsis thaliana* and so on (Martin et al., 2006; Brauer et al., 2011; Goodall et al., 2013; Konishi et al., 2014). *AtGLN1.2*, *AtGLT1* and *HvGS1.3* were highly expressed and induced by ammonium (Goodall et al., 2013; Konishi et al., 2014). Overexpression of *OsGS1* improves spikelet yield and N-use efficiency in rice (Brauer et al., 2011). Similarly, overexpression of *ZmGS1* increases maize grain yield by 45% (Martin et al., 2006). Consistent with the ammonium-induced expressions of *XsiGLN1.1*, *XsiGLN1.3* and *XsiGLN1.4*, leaf and root GS activities increased for *X. sibiricum* with increasing environmental ammonium to nitrate ratio, and were always higher than those of *X. strumarium* under high ammonium to nitrate ratio (**Figure 8**).

Among the eleven ammonium-induced *GOGAT* transcripts in *X. sibiricum*, *XsiGLT1b* (followed by *XsiGLU1* and *XsiGLT1h*) were the most highly expressed (**Figure 4D**), which may contribute to ammonium assimilation, and thus to growth of *X. sibiricum*. Chichkova et al. (2001) found that high expression levels of *GOGAT* genes improve N assimilation in tobacco. Overexpression of *OsGOGAT* improves grain filling in rice (Yamaya et al., 2002). In contrast, knockout of *OsNADH-GOGAT1* gene reduces total amino acid content and yield in rice (Tamura et al., 2010).

For *X. sibiricum*, much more DETs were induced by ammonium than by nitrate (**Figure 1**). Yang et al. (2018) also found that ammonium-induced DETs were 1.5 times higher than nitrate-induced DETs in tea tree, which also prefers ammonium compared with nitrate. In addition, the number of the DETs enriched in biosynthesis of amino acids pathways was much greater than that enriched in any other 11 unique pathways for *X. sibiricum*, and most of the DETs enriched in this pathway were induced by ammonium (**Figures 2**, **S7**). The biosynthesis of histidine, phenylalanine, glutamate, glutamine, proline, and arginine may be enhanced by ammonium (**Figure S7**). For *X. strumarium*, however, DETs were not significantly enriched in this pathway (**Figure 2**). It has been reported that the concentrations of glutamate, glutamine, and asparagine in rice and *Myriophyllum aquaticum* increase with the increase of ammonium treatment concentration and time (Yang et al., 2017; Zhang et al., 2021). However, N storage in glutamate (C/N = 5/1), glutamine (C/N = 2.5/1) and asparagine (C/N = 2/1) may cause carbohydrate deficiency and inhibit plant growth (Bittsanszky et al., 2015). In order to reduce carbon consumption, N can also be stored in arginine (C/N = 1.5/1), which had the lowest C/N ratio among all amino acids. Thus, the fast amino acid synthesis and conversion may enhance the ability to use ammonium for *X. sibiricum*.

Our results also indicate that *X. sibiricum* and *X. strumarium* may share some similar strategies to acclimate different N forms. Sixty percent of the top 30 pathways with DETs significantly enriched were common in these two species, including ascorbate and aldarate metabolism pathways (**Figure 2**). In this pathway, ascorbate peroxidase and ascorbate are involved in reactive oxygen species (ROS) quenching, and therefore decrease oxidative damage to plants in ammonium. It has been found that plants can accumulate ammonium in different tissues, which are seriously harmful to plants (Apel and Hirt, 2004).

### Gibberellin and its functions

Our research found five ammonium-induced GA biosynthesis and metabolism related DETs in *X. sibiricum*. Among them, *XsiGA3OX1a* was the most highly expressed, followed by *XsiGA2OX2* and *XsiGA2OX1b* (**Figure 4A**). It was reported that GA3OX was participated in biosynthesis of GA1 and GA4, which are two main active forms of GA (Yamaguchi et al., 1998; Ueguchi-Tanaka et al., 2007). The induction of *XsiGA3OX1a* may enhance the contents of GA1 and GA4 in ammonium for *X. sibiricum*. Supplement of exogenous GA1 or GA4 up regulated expressions of all ammonium-utilization related transcripts tested in *X. sibiricum*, including *XsiAMT1.1a*, *XsiGLN1.1* and *XsiGLT1b* (**Figure 6**), and the effects of GA4 were higher than those of GA1, indicating that GA4 play more important roles in ammonium-utilization. GA4, as well as other bioactive forms of GA, play critical roles in many developmental processes, including seed germination, root elongation, flowering, and fruit development (Hedden and Sponsel, 2015; Binenbaum et al., 2018). Dai et al. (2016) also found that GA influenced invasion success of the exotic plant *Wedelia trilobata*. However, few studies explore the roles of GA in utilization of ammonium, and the regulations of N forms on expression of GA biosynthesis and metabolism related transcripts is still unknown. Recently, it was reported that growth-regulating factor 4 (*GRF4*), which is involved in GA signal pathway, can improve ammonium uptake and rice growth (Li et al., 2018). ChIP–PCR showed that *GRF4* can bind to the promoter regions of *OsAMT1.1* and *OsGS1.2* (Li et al., 2018). Our results provided direct evidence that GA, especially GA4, is involved in regulations of ammonium uptake and assimilation.

In addition, some DETs were also significantly enriched in the pathway of zeatin biosynthesis for *X. sibiricum* but not for *X. strumarium* (**Figure 2**). tRNA dimethylallyl transferase [EC:2.5.1.75] and cis-zeatin O-glucosyl transferase [EC:2.4.1.215] were induced by ammonium (**Figure S8**). These results indicate that different forms of zeatin may also be involved in the stronger ability to use ammonium for *X. sibiricum* relative to *X.strumarium*.

## Conclusion

*Xanthium sibiricum* increases expressions of many transcripts related with ammonium uptake and assimilation in response to ammonium, improving ammonium uptake, assimilation and thus growth. *XsiAMT1.1a*, *XsiGLN1.1*, *XsiGLT1b*, *XsiGA3OX1a*, *XsiGA3OX1b*, *XsiGA2OX2* as well as others may be important for the strong ability to use ammonium in *X. sibiricum*. Gibberellin, especially GA4, is involved in the regulation of ammonium utilization. Ammonium regulates expressions of some transcripts related with GA biosynthesis and metabolism, which in turn induces expressions of transcripts related with ammonium uptake and assimilation. Zeatins may also be involved in the regulation of ammonium utilization in *X. sibiricum*. Our study elucidates the molecular mechanisms with which the *Xanthium* species acclimate to different soil nitrogen forms, contributing to understanding the invasion mechanisms of *X. strumarium* and its invasion habitat selection.

## Data availability statement

The datasets presented in this study can be found in online repositories. The names of the repository/repositories and accession number(s) can be found below: https://www.ncbi.nlm.nih.gov/, PRJNA751368 https://www.ncbi.nlm.nih.gov/, PRJNA784152.

## Author contributions

CZ, Y-LF: Conceived the ideas and designed methodology. CZ, J-JL, J-BZ, ZZ, S-TW: Conducted the experiment. CZ, J-JL, X-JZ, T-SF: Analyzed the data. CZ, Y-LF: Wrote the manuscript. All authors contributed to the article and approved the submitted version.

## Funding

This study was supported by the National Natural Science Foundation of China (31971557, 31670545 and 32001235).

## Conflict of interest

The authors declare that the research was conducted in the absence of any commercial or financial relationships that could be construed as a potential conflict of interest.

## Publisher’s note

All claims expressed in this article are solely those of the authors and do not necessarily represent those of their affiliated organizations, or those of the publisher, the editors and the reviewers. Any product that may be evaluated in this article, or claim that may be made by its manufacturer, is not guaranteed or endorsed by the publisher.
