# Supplementary material for: Transcripts related with ammonium use and effects of gibberellin on expressions of the transcripts responding to ammonium in two invasive and native Xanthium species
Source: Front Plant Sci. 2022 Oct 26;13:1035137. doi: 10.3389/fpls.2022.1035137 (PMC9644049; doi:10.3389/fpls.2022.1035137)
Supplement: Supplementary file 1 [file DataSheet_1.docx]

Table S1 Primers used in this study

Primer sequences

*XsiActin* U GCGACATCAAGGAGAAGC

*XsiActin* L CGAGGAAAGAGGGTTGGA

*XsiGA3OX1a* U GACCTCCTTCACATACTATCC

*XsiGA3OX1a* L CCAAGATACTCGCTCCAAG

*XsiAMT1.1a* U CTAGCAGCTCAGATCATCC

*XsiAMT1.1a* L CGTCATATCCATTCCTTGTAG

*XsiGLN1.2* U TTGCCTGTATGCTGGTATT

*XsiGLN1.2* L GCTATCTCAGTAATCCTCTCA

*XsiGLT1a* U CTTCCTCGGTCCAGAATC

*XsiGLT1a* L TCTACGCTCGTCAAGAAC

Table S2 Statistics of the read alignments in the RNA-Seq study

Sample length Reads Bases Q20 (%) Q30 (%) GC (%) N (ppm)

1-1 150.00 40745000 6111750000 96.92 92.47 42.55 46.48

1-2 150.00 43711712 6556756800 96.98 92.58 42.75 45.81

1-3 150.00 41521892 6228283800 96.95 92.51 42.69 46.07

2-1 150.00 42396384 6359457600 97.01 92.65 43.16 46.61

2-2 150.00 63817064 9572559600 96.98 92.60 42.99 46.29

2-3 150.00 39930228 5989534200 96.42 91.40 43.06 41.87

3-1 150.00 61887520 9283128000 96.10 90.92 43.01 15.51

3-2 150.00 41076536 6161480400 95.78 90.01 42.98 14.90

3-3 150.00 43980950 6597142500 96.16 90.85 42.97 26.56

4-1 150.00 47641906 7146285900 95.89 90.29 43.30 24.26

4-2 150.00 43012328 6451849200 95.81 90.08 43.17 14.71

4-3 150.00 42510974 6376646100 96.05 90.63 42.93 24.05

Samples 1 and 2 represent data for *Xanthium sibiricum* under ammonium-N and nitrate-N, respectively. Samples 3 and 4 represent data for *X. strumarium* under ammonium-N and nitrate-N, respectively. The numbers (1, 2 and 3) after “-” represent three independent biological replicates for each treatment.

**Fig. S1** GO terms enrichment analysis for differently expressed transcripts in *Xanthium sibiricum* (a) and *X. strumarium* (b). Pink, green and blue columns indicate molecular function, cellular component and biological process, respectively.

**Fig. S2** Phylogenetic analyses of the differently expressed transcripts (DETs) of gibberellin 2beta-dioxygenase (GA2ox), gibberellin 3beta-dioxygenase (GA3ox) and gibberellin-44 dioxygenase (GA20ox) in *Xanthium sibiricum* and *X. strumarium*. The analyses were performed using MEGA6. The sequences of *X. sibiricum* (in red) and *X. strumarium* (in blue) were from our PacBio sequencing; those of *Arabidopsis thaliana* (in yellow) were from TAIR. Different letters after the same gene represent different transcripts of the gene.

**Fig. S3** Phylogenetic analyses of the differently expressed transcripts (DETs) of ammonium transporter (AMT) in *Xanthium sibiricum* and *X. strumarium*. For *X. strumarium*, there were no DETs of ammonium transporter. The analyses were performed using MEGA6. The sequences of *X. sibiricum* (in red) and *X. strumarium* (in blue) were from our PacBio sequencing; those of *Arabidopsis thaliana* (in yellow) were from TAIR. Different letters after the same gene represent different transcripts of the gene.

**Fig. S4** Phylogenetic analyses of the differently expressed transcripts (DETs) of glutamine synthetase (GLN) in *Xanthium sibiricum* and *X. strumarium*. The analyses were performed using MEGA6. The sequences of *X. sibiricum* (in red) and *X. strumarium* (in blue) were from our PacBio sequencing; those of *Arabidopsis thaliana* (in yellow) were from TAIR. Different letters after the same gene represent different transcripts of the gene.

**Fig. S5** Phylogenetic analyses of the differently expressed transcripts (DETs) of glutamate synthase (GLT and GLU) in *Xanthium sibiricum* and *X. strumarium*. The analyses were performed using MEGA6. The sequences of *X. sibiricum* (in red) and *X. strumarium* (in blue) were from our PacBio sequencing; those of *Arabidopsis thaliana* (in yellow) were from TAIR. Different letters after the same gene represent different transcripts of the gene.

**Fig. S6** Differently expressed transcripts (DETs) of *Xanthium strumarium* enriched in DNA replication pathway. Red represents that this kind of DETs were induced by nitrate.

**Fig. S7** Differently expressed transcripts (DETs) of *Xanthium sibiricum* enriched in biosynthesis of amino acids pathway. Blue represents that this kind of DETs were significantly induced by ammonium. Red represents that this kind of DETs were significantly induced by nitrate. Green represents that part of this kind of DETs were significantly induced by ammonium, and others were significantly induced by nitrate.

**Fig. S8** Differently expressed transcripts (DETs) of *Xanthium sibiricum* enriched in zeatin biosynthesis pathway. Blue represents that this kind of DETs were significantly induced by ammonium. Green represents that part of this kind of DETs were significantly induced by ammonium, and others were significantly induced by nitrate.
